# A young woman from an *Echinococcus*-endemic area with progressive abdominal distension: a case report

**DOI:** 10.1186/s13256-020-02388-8

**Published:** 2020-06-08

**Authors:** Albrecht Boehlig, Valentin Blank, Thomas Karlas, Henning Trawinski, Hans-Michael Hau, Andri Arnosson Lederer, Thomas Berg

**Affiliations:** 1 Divison of Hepatology, Clinic of Gastroenterology, Hepatology, Infectious Diseases and Pneumology, University Hospital Leipzig, Liebigstraße 20, 04103 Leipzig, Germany; 2 Clinic of Gastroenterology, Hepatology, Infectious Diseases and Pneumology, University Hospital Leipzig, Leipzig, Germany; 3 Integrated Research and Treatment Center AdiposityDiseases Leipzig, Faculty of Medicine, University of Leipzig, Leipzig, Germany; 4 Division of Infectiology and Tropical Medicine, Clinic of Gastroenterology, Hepatology, Infectious Diseases and Pneumology, University Hospital Leipzig, Leipzig, Germany; 5 Divison of Hepatobiliary and Transplant Surgery, Department of Visceral, Transplant, Thorax and Vascular Surgery, University Hospital Leipzig, Leipzig, Germany

**Keywords:** Cystic tumor, *Echinococcusgranulosus*, Hydatid disease, Ovarian cystadenoma, Case report

## Abstract

**Background:**

Cystic echinococcosis is a zoonotic infection caused by *Echinococcus granulosus*. This case report shows the difficulty in differential diagnosis in a patient with highly suspected hydatid disease.

**Case presentation:**

A 29-year-old Chinese woman presented with progressive abdominal distension. Imaging results revealed a large multicystic tumor with typical features of hydatid disease. There was no clear relationship between the cystic tumor and the liver, which led to the assumption of primary extrahepatic cystic echinococcosis. After albendazole therapy was initiated, a laparotomy was performed and a huge ovarian cystadenoma was diagnosed.

**Conclusions:**

This case highlights the possible challenges of differential diagnosis in patients with suspicion of hydatid cysts.

## Background

Hydatid disease is a zoonotic infection caused by cestode species from the genus *Echinococcus*. The cystic form of echinococcosis is caused by *Echinococcus granulosus* [1]. Both the cystic and the alveolar forms of echinococcosis are endemic in Western China, which has one of the highest prevalence rates of up to 6.8% [2, 3]. The diagnosis relies mainly on imaging results. Therapeutic options range from percutaneous interventions, surgery, and antihelminthic therapy to “watch and wait” strategies. Although echinococcosis is a preventable and treatable disease, echinococcosis continues to be a major public health problem in many countries [4]. This case report aims to demonstrate the case of a patient from an *Echinococcus*-endemic area with highly suspected hydatid disease and an unexpected outcome with a challenging differential diagnosis.

## Case presentation

In May 2019, a 29-year-old Chinese woman presented to her physician because of progressive abdominal pressure and loss of appetite over the last 3 months. She complained of lack of strength and could only eat a small portion of meals because her abdomen felt increasingly distended. No fever or enlarged lymph nodes were reported. In addition, her weight increased by 3 kg in the last 9 months despite reduced food intake. Her past medical history was unremarkable. Her heritage is the western part of China. At presentation, she had been living in Germany for 5 years and reported no trips abroad within the last 6 months. Her last visit to China was in 2016. She is currently a student at university.

A physical examination showed a slightly distended abdomen with pressure pain in the right upper quadrant and very scarce bowel sounds. Laboratory results revealed only slight elevation of aspartate aminotransferase (0.72 μkat/l; reference range 0.17–0.6); all other liver enzymes were within normal range. There were no pathologic levels of white blood cells (5.0 × 10^9^/l; reference range 3.5–9.8), lymphocytes (1.61 × 10^9^/l; reference range 1–2.9), eosinophils (0.09 × 10^9^/l; reference range 0–0.5), and neutrophils (2.92 × 10^9^/l; reference range 1.6–7.1) and liver function tests, such as bilirubin (10.5 μmol/l; reference range < 17.1), albumin (50.6 g/l; reference range 35–52) and international normalized ratio (1.0). Furthermore, there were no elevated inflammatory parameters (C-reactive protein 2.32 mg/l; reference range < 5) and normal values for tumor markers such as alpha-fetoprotein, carbohydrate antigen 19-9, and carcinoembryonic antigen.

**Fig. 1:**
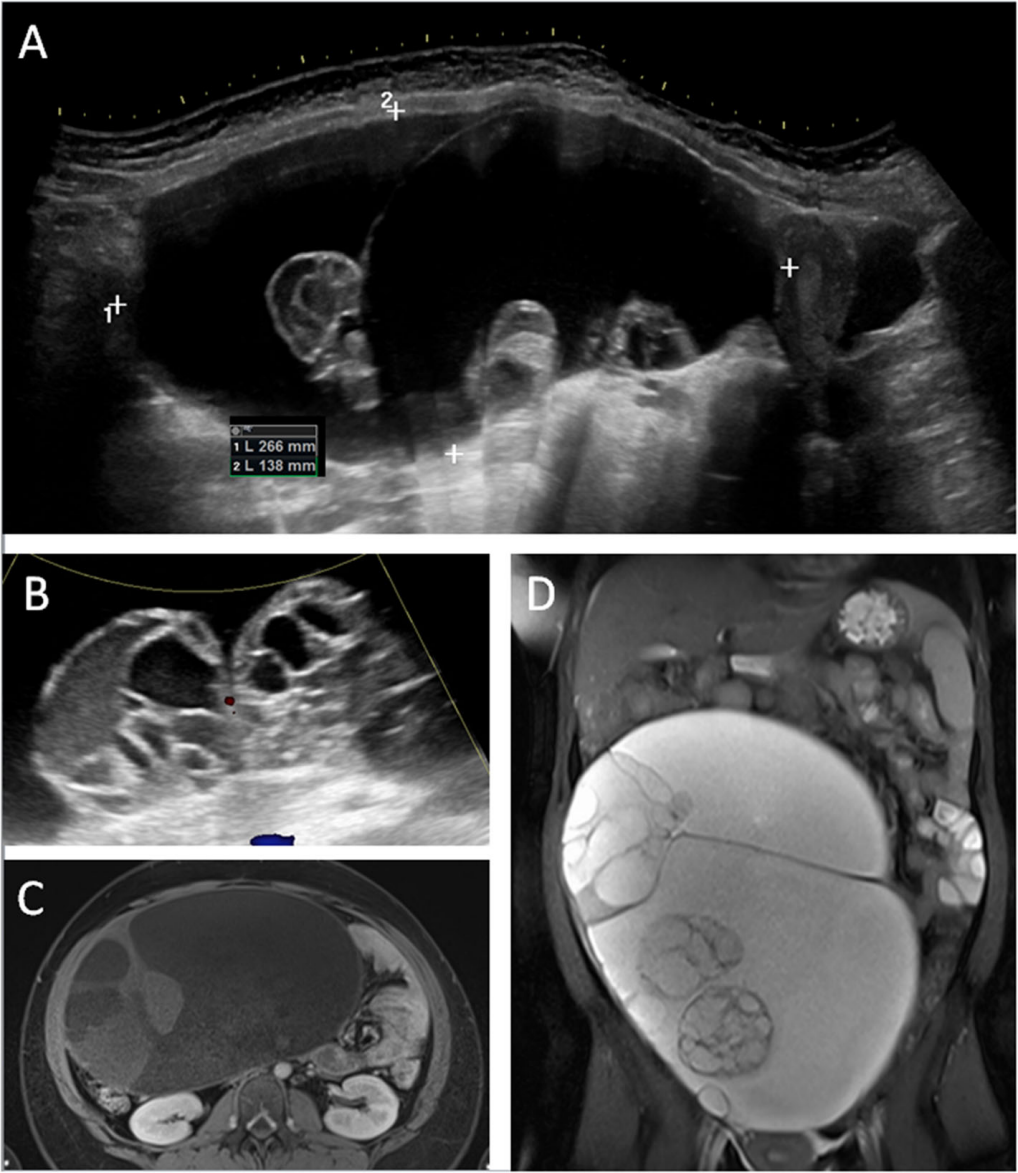
**a** Abdominal ultrasound (panorama mode in sagittal plane) revealing the large cystic mass filling nearly the whole abdomen with a cranio-caudal diameter of approximately 27 cm. The main cystic tumor consists of many smaller cyst-in-cyst structures. **b** Ultrasound image with the typical cyst-in-cyst sign suspicious for diagnosis of hydatid disease in stage CE2. **c, d** Magnetic resonance imaging scan of the abdomen (**b**, T1 sequence axial; **c**, T2 sequence coronal) showing the marked-off large cystic mass filling nearly the whole abdomen and displacing parenchymal organs and intestinal structures

An abdominal ultrasound revealed a huge polycystic formation with cyst-in-cyst configuration filling nearly her whole abdomen. The whole tumor-like mass was approximately 27 × 14 × 23 cm (see Fig. 1a, b). Due to the abdominal mass, the liver parenchyma was displaced cranially to diaphragm and could only be investigated by a transcostal view. Therefore, the incomplete liver examination could neither prove nor exclude further liver cysts. There was no sharp delineation between the abdominal cystic formation and the liver parenchyma. Furthermore, there was no evidence of another originating organ association of the huge cystic mass. Relying on clinical and imaging results, the diagnosis of hydatid disease (infection by *E. granulosus*) was suspected and staged as CE2, which refers to an active infection with many cyst-in-cyst lesions according to the World Health Organization (WHO) classification [4]. Consequently, we performed an Enzyme-linked Immunosorbent Assay (ELISA) test for *Echinococcus* species, which turned out to be negative. Due to the capsule isolation of the parasite from the host’s immune system, serology tests, such as indirect hemagglutination and ELISA, may be negative in approximately 20% of cases [5], especially in extrahepatic manifestations. In addition, false negative serology test results have been reported in our center in cases with immunosuppression [6].

Due to the highly suspected diagnosis of hydatid disease, we initiated an antihelminthic therapy with albendazole 2 × 400 mg per day prior to planned intervention to prevent spread of hydatid cysts in other organs. In particular, under consideration of the risk of cyst rupture and the increasing symptoms of our patient due to progressive compression, a further diagnostic workup was performed in June 2019 prior to the scheduled surgery. We refrained from doing a biopsy of the cysts in advance to confirm the diagnosis. Transvaginal and transabdominal gynecologic sonography revealed normal-sized ovaries, but the right ovarium was of limited visibility. A magnetic resonance imaging (MRI) scan which was performed to rule out cysto-biliary fistulas did not show a distinct relationship between the cystic tumor and the liver, which confirmed the ultrasound findings and led to the assumption of primary extrahepatic cystic echinococcosis (see Fig. 1c, d). A preoperative chest X-ray showed no signs of pulmonary cysts.

In July 2019, a median laparotomy was performed and intraoperatively the cystic tumor appeared to derive from the right ovary. In consequence, a right adnexectomy was performed and multiple biopsies and cytological examinations from the peritoneum were done. The explanted cystic tumor turned out to be a multilocular mucinous cystadenoma weighing 6135 g originating from the right ovary. Histology revealed an intestinal subtype and the remaining ovarian tissue showed only slight fibrotic changes. The remaining left ovary showed no abnormalities and was kept *in situ* because of the childbearing age of our patient. As a consequence of the histologic result, we stopped albendazole therapy. A follow-up gynecological examination in the postoperative period showed no significant pathological findings. Eventually, she recovered well from surgery and was discharged on the seventh postoperative day. She was referred to her local gynecologist for regular follow-up visits. Retrospectively, this ovarian tumor was not suspected in the preoperative gynecological workup.

## Discussion and conclusions

This case report highlighted an unexpected outcome of an ovarian cystadenoma in a patient with highly suspected hydatid disease.

Ovarian mucinous cystadenoma is the most common ovarian neoplasm responsible for 20% of all ovarian tumors. They are mostly benign in 80% of cases and are commonly asymptomatic at early stages. They origin from mucin-producing epithelial cells and their average diameter ranges from 15 to 30 cm [7]. Such ovarian tumors can grow to a very large size with heavy weight, which has been demonstrated in a case report from the early twentieth century showing a multicystic ovarian tumor with a weight of nearly 149 kg [8].

Typical organ manifestations of hydatid disease involve the liver (75%) and the lungs (15%). Less frequently, cysts can be situated in other abdominal organs and the abdominal cavity [9–11]. Primary peritoneal hydatidosis accounts for just 2% of all intra-abdominal hydatid diseases [12]. In such cases, the diagnostic workup can be challenging and may cause diagnostic delay. Involvement of the ovary is seen very rarely and can be secondary due to peritoneal spread of daughter cysts after rupture of a liver hydatid cyst [13]. In particular, in cases with a history of *Echinococcus* exposure in endemic areas, imaging findings may be misleading. Our case clearly illustrates that even a cyst-in-cyst configuration, which is a very typical sign of active *Echinococcus* infection [4], may also derive from ovarian lesions, especially from cystadenoma or cystadenocarcinoma. In particular, in women of reproductive age, hydatid cysts may be unilocular and can mimic ovarian cystadenoma [13]. Therefore, a careful consideration of imaging findings is required and a biopsy should be considered in unclear cases.

In conclusion, imaging features have a low specificity in the diagnosis of hydatid disease and cystic ovarian cystadenoma may represent one of the less well-described potential differential diagnoses, especially in cases with exclusive extrahepatic manifestations.

## Data Availability

Not applicable.
